# Assessing Training Zones in Adult Men with Obesity: A New Field Test

**DOI:** 10.3390/jfmk11020202

**Published:** 2026-05-21

**Authors:** Mattia D’Alleva, Luca Innella, Nicola Giovanelli, Lara Mari, Jacopo Stafuzza, Simone Zaccaron, Francesco Graniero, Véronique Billat, Enrico Rejc, Stefano Lazzer

**Affiliations:** 1 Department of Theoretical and Applied Sciences, eCampus University, Novedrate, 22060 Como, Italy; mattia.dalleva@uniecampus.it; 2 Department of Medicine, University of Udine, 33100 Udine, Italy; info@nexthillcoaching.com (N.G.); mari.lara@spes.uniud.it (L.M.); jacopo.stafuzza@uniud.it (J.S.); simone.zaccaron@uniud.it (S.Z.); enrico.rejc@uniud.it (E.R.); 3 School of Sport Sciences, University of Udine, 33100 Udine, Italy; 4 Department of Biological and Environmental Sciences and Technologies (DiSTeBA), University of Salento, 73100 Lecce, Italy; innellaluca@gmail.com; 5 Department of Neurosciences, Biomedicine and Movement Sciences, University of Verona, 37129 Verona, Italy; 6 Physical Exercise Prescription Center, Azienda Sanitaria Universitaria Friuli Centrale, 33013 Gemona del Friuli, Udine, Italy; francesco.graniero@asufc.sanita.fvg.it; 7 Unité de Biologie Intégrative des Adaptations à l’Exercice, Université Evry Paris-Saclay, 91000 Evry-Courcouronnes, France; veroniquelouisebillat@gmail.com; 8 BillaTraining SAS, 32 rue Paul Vaillant-Couturier, 94140 Alforville, France

**Keywords:** RABIT^®^ test, obesity, aerobic threshold, anaerobic threshold, exercise prescription

## Abstract

**Background:** The aim of this study was to investigate the preliminary evidence supporting the validity of the Running Advisor Billat Training test (RABIT^®^) in determining the three intensity domains in male adults with obesity. **Methods:** Thirteen male adults with obesity completed a graded (GRAD) and a RABIT^®^ test. The RABIT^®^ test consisted of three fixed levels of perceived exertion (RPE): (1) 10 min at RPE 13, (2) 5 min at RPE 16, and (3) 3 min at RPE 18. GRAD was composed of 1 min step, increasing speed by 0.5 km/h every minute until volitional exhaustion. **Results:** At RPE 18, maximal oxygen consumption ($\dot{V}$O_2_max), minute ventilation ($\dot{V}$_E_), maximal heart rate (HRmax), and running speed were not significantly different from the values measured during the GRAD. As well, oxygen consumption ($\dot{V}$O_2_), $\dot{V}$_E,_ and HR measured during RPE 16 and RPE 13 of the RABIT^®^ test were not significantly different from the anaerobic threshold (AnT) and aerobic Threshold (AerT) values measured during GRAD. However, running speed at RPE 16 and RPE 13 of the RABIT^®^ test was lower by −5.03% (*p* = 0.041) and −7.00% (*p* < 0.001), compared to GRAD. **Conclusions:** The data obtained in our study provide preliminary evidence supporting the ability of the RABIT^®^ test to estimate maximal exercise parameters, as well as most parameters associated with AerT and AnT. Consequently, the test may be useful for identifying the three training intensity domains and for planning training sessions for adults with obesity.

## 1. Introduction

Obesity is recognized as one of the most important global public health challenges and currently ranks as the fourth leading cause of death worldwide [1]. This trend is closely linked to sedentary behaviors, whereas consistent evidence shows that physical activity provides significant health benefits in adults with obesity [2,3]. In obesity management, both team-based recreational sports [4] and individual activities such as running [5] offer multiple benefits across psychosocial, physical, and metabolic domains, while also promoting longevity [6,7]. In this regard, one of the key parameters in exercise prescription is exercise intensity, which plays a central role in guiding targeted interventions, monitoring training, and ensuring progressive adaptation to training stimuli [8]. In endurance sports, maximal oxygen consumption ($\dot{V}$O_2_max) and ventilatory thresholds are among the primary physiological parameters used for exercise intensity prescription [9]. The gold-standard assessment is the graded exercise test (GRAD), which determines ventilatory thresholds and $\dot{V}$O_2_max based on individual physiological characteristics [10]. Submaximal and maximal gas exchange measurements obtained during the GRAD are used to identify three physiological training zones (i.e., exercise intensity domains) for prescribing training intensity [8]: zone 1 (z1) corresponds to speeds or heart rate (HR) below the aerobic threshold (AerT); zone 2 (z2) includes values between the two ventilatory thresholds; and zone 3 (z3) corresponds to speeds or HR between the anaerobic threshold (AnT) and $\dot{V}$O_2_max [11,12]. However, these laboratory-based assessments require specific technical expertise, dedicated facilities, and costly equipment, which limits their feasibility in field settings. To overcome these limitations, several field-based tests have been developed. For instance, the Cooper and 6 min tests were designed to estimate $\dot{V}$O_2_max [13,14]; other field protocols were developed to calculate critical speed (CS) [15], and submaximal protocols have been proposed to estimate AerT [16]. Nevertheless, these tests (i) estimate only one variable at a time and (ii) do not provide a comprehensive overview of the different intensity domains. To address the limitations described above, the Running Advisor Billat Training (RABIT^®^) test was developed as a simple method to identify submaximal and maximal training zones in recreational runners [17]. This test evaluates speed and HR at AerT, AnT, and $\dot{V}$O_2_max using the Borg Rating of Perceived Exertion (RPE) scale (6–20 points) [17,18]. The protocol consists of three stages of 10, 5, and 3 min at progressively increasing RPE levels (11, 14, and 17), with 1 min passive recovery intervals between stages [17]. In healthy adults, RPE is closely associated with exercise intensity and reflects how “hard” or “easy” a physical task is perceived [19]. When anchored to ventilatory thresholds during a GRAD or to blood lactate concentrations of 2.0 and 4.0 mmol L^−1^ (i.e., corresponding to lactate thresholds) [20,21], RPE values can be used to define the boundaries between moderate, heavy, and severe intensity domains [20]. However, for application in individuals with obesity, the RABIT^®^ test may require adaptation. In this population, RPE values are generally higher at the same absolute workload due to the increased metabolic cost of locomotion, reduced mechanical efficiency, and greater cardiovascular and thermoregulatory strain [22,23]. Accordingly, several authors have reported different RPE values corresponding to the two ventilatory thresholds and $\dot{V}$O_2_max in different populations (e.g., obese and non-athletic individuals) [11,20,24]. Specifically, AerT has been observed at approximately RPE ≤ 13 [20,24,25], AnT at RPE 14–16 [20,24,25], and $\dot{V}$O_2_max at RPE ≥ 17 [11,25,26]. Nevertheless, in most studies involving adults with obesity, training intensity has been prescribed according to a fixed percentage of maximal heart rate (HRmax) or $\dot{V}$O_2_max [13], whereas ventilatory thresholds provide a more individualized approach for optimizing training adaptations [8]. Based on this evidence and considering the specific characteristics of the studied population, the aim of this study was to investigate the preliminary evidence supporting the validity of a modified version of the RABIT^®^ field test for identifying the three physiological training zones in individuals with obesity, without the need for expensive equipment. The protocol was adapted by adjusting RPE levels to 13, 16, and 18 while maintaining the original stage durations in order to provide a specific tool for exercise prescription in obesity.

## 2. Materials and Methods

### 2.1. Participants

Thirteen male adults with obesity (mean age of 40.2 ± 4.1 years; mean body mass [BM] of 94.2 ± 10.2 kg; mean body mass index [BMI] of 30.9 ± 4.4 kg∙m^−2^, and mean fat mass [FM] of 34.3 ± 6.4%) were enrolled in the study. All subjects had a complete medical history and underwent a physical examination. BMI was stable during the previous two months. All the volunteers were moderately physically active (i.e., performed continuous aerobic activity for longer than 20 min two or three times/week) based on the International Physical Activity Questionnaire Short Form (IPAQ-SF) [27]. All participants were healthy, had no current injuries, and were not taking medications regularly or using drugs known to affect energy metabolism. Participants were informed of the protocol, and written informed consent was obtained. A priori, we assumed a minimum clinically relevant difference in $\dot{V}$O_2_max between the RABIT^®^ and the incremental treadmill test of 3.0 mL·kg^−1^·min^−1^, along with an expected standard deviation of the within-subject difference of 6.4 mL·kg^−1^·min^−1^ [18]. This corresponds to an effect size of d_n_ = 0.47 with a sample of 13 participants; therefore, the findings should be interpreted as exploratory or pilot in nature.

### 2.2. Experimental Design

The study was approved by the Ethics Committee of the Friuli–Venezia–Giulia Region (Italy) (protocol number 1764). Before study initiation, the protocol, aims, and procedures were carefully explained to all participants, who provided written informed consent. Participants completed the GRAD and RABIT^®^ tests in a randomized, counterbalanced order, separated by 5–7 days of rest. The RABIT^®^ test was not designed to replace the GRAD, which remains the objective and standardized reference method. Rather, it was developed as a practical, perception-based tool to help identify training intensity domains in adults with obesity, particularly in settings where administration of the GRAD may be less feasible. They were instructed to refrain from strenuous exercise for 24 h prior to each testing session and to wear the same running shoes in each test. Advanced footwear technology was not permitted due to its ergogenic effect on oxygen consumption at submaximal intensity [28]. All tests were performed at the same time of day on a 400 m outdoor track (Gemona del Friuli, Udine, Italy), under medical supervision and comparable environmental conditions (12.4 ± 3.1 °C; 51.3 ± 9.8% relative humidity). During both visits, the standardized warm-up consisted of 15 min of easy running followed by three 10 s sprints, each separated by 60 s of passive recovery. After the warm-up, participants were equipped with a portable metabolic system (K5, Cosmed, Rome, Italy) to record cardiorespiratory parameters. All participants had taken part in a previous study [29] and had been trained in the use of the Borg 6–20 RPE scale. In that study, as well as during the GRAD phase of the current protocol, they reported their RPE during the final minute of each stage by marking the scale and provided a session-RPE at the end of each exercise session. Therefore, they entered the present study with documented familiarity with the instrument [29]. During the RABIT^®^, the RPE scale was administered every 200 m. Data collection was carried out by graduates in Sport and Exercise Sciences who were trained on the use of the metabolic system and in administering the Borg 6–20 scale, following standardized procedures.

### 2.3. Measurements

#### 2.3.1. Graded Exercise Test (GRAD)

During the GRAD, all the participants were instructed to follow a collaborator with the bike for pacing. The starting speed was ~70% of the speed of their 10.000 m previously run on a 400 m track. The duration of each step was 1 min, and the speed increased by 0.5 km h^−1^ every minute until volitional exhaustion. The number of steps completed was between 10 and 15. Oxygen uptake ($\dot{V}$O_2_), carbon dioxide production ($\dot{V}$CO_2_), minute ventilation ($\dot{V}$_E_), and heart rate (HR) were measured during this test using a wearable metabolic unit (K5, Cosmed, Roma, Italy) and a chest strap (Garmin HRMrun, Olathe, KS, USA), respectively. We calibrated the volume and gas analyzers before each test using a 3 L calibration syringe and calibration gas (16.00% O_2_ and 5.00% C’O_2_), respectively. We determined the AerT and AnT with the V-slope method [30]. $\dot{V}$O_2_max was calculated as the average 30 s $\dot{V}$O_2_ according to previously established criteria [31]: (i) plateau in $\dot{V}$O_2_ (i.e., increase < 150 mL min^−1^), (ii) respiratory exchange ratio (RER) > 1.1, and (iii) ≥ 90% of theoretical HRmax.

#### 2.3.2. RABIT^®^ Test

**Figure 1:**
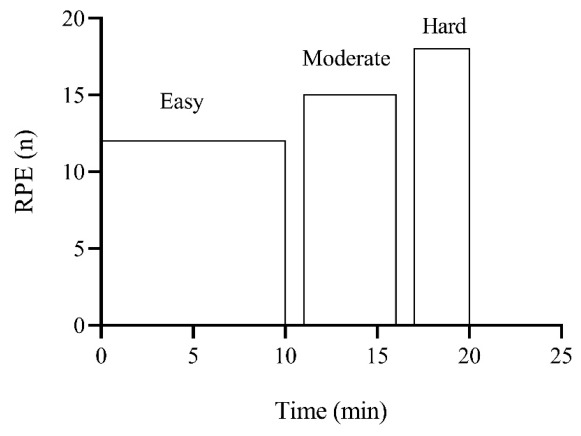
Schematic representations of the RABIT^®^ test. Easy: running for 10 min at 13 on the Borg scale. Moderate: running for 5 min at 16 on the Borg scale. Hard: running for 3 min at 18 on the Borg scale. Between each step, subjects rested for 1 min.

The test included three incremental exercise stages: 10 min at RPE 13, 5 min at RPE 16, and 3 min at RPE 18 (Figure 1). Each step was followed by a 1 min standing rest period. Participants were instructed to ‘hold’ the target RPE and change their running speed according to the given RPE. The RPE scale could be viewed by participants at regular intervals (i.e., every 200 m). We asked participants to run without a watch to avoid external influences. Throughout the RABIT^®^ test, the volunteers wore the wearable metabolic unit (K5, Cosmed, Roma, Italy) and a chest strap (Garmin HRMrun, Olathe, KS, USA) to collect cardiorespiratory and ventilatory parameters, pacing response, and HR. Then, we averaged the data from the last minute of each step and compared the first step (RPE 13) with AerT, the second step (RPE 16) with AnT, and the third step (RPE 18) with maximum values.

### 2.4. Statistical Analysis

The data were analyzed using GraphPad Prism (version 9.4.0), with significance set at *p* < 0.05. All parameters ($\dot{V}$O_2_, RER, $\dot{V}$_E_, HR, and running speed) are expressed as means and standard deviations (SDs) for the GRAD and RABIT^®^ test. The Shapiro–Wilk test was used to evaluate the normality of the data. A Greenhouse–Geisser correction was used in cases of sphericity assumption violations. Only for HR analyses, we used data from 9 participants due to a technical problem with the chest strap (Garmin HRMrun, Olathe, KS, USA). $\dot{V}$O_2_, RER, $\dot{V}$_E_, HR, and speed measured during the RABIT^®^ test at RPE 13, 16, and 18 were compared with AerT, AnT, and $\dot{V}$O_2_max, respectively, obtained during GRAD. A paired *t*-test was used to compare the results of RABIT^®^ and the GRAD. Identification of the slow component of HR during the RABIT^®^ test at RPE 16 was done using a linear regression line and correlation analysis by the method of least squared residuals [32]. The Bland–Altman test was used to verify the parameters obtained during RABIT^®^ and GRAD, as this method provides a robust assessment of agreement between measurement techniques, allowing identification of systematic bias and the limits of agreement [33]. Pearson’s coefficient was used for the correlation between RABIT^®^ and GRAD. The correlation was classified as low (r = 0.30–0.50), moderate (r = 0.50–0.70), high, and very high (r = 0.70–1.00) [34]. The predictive accuracy of the RABIT^®^ parameters was set within ± 5% of GRAD parameters and is reported as percentage values [35]. Effect sizes (ESs) were calculated between the parameters of the RABIT^®^ and GRAD using Cohen’s d (0 < d < 0.20 small; 0.20 < d < 0.50, medium; 0.50 < d, large).

## 3. Results

### 3.1. Maximum Parameters

**Figure 2:**
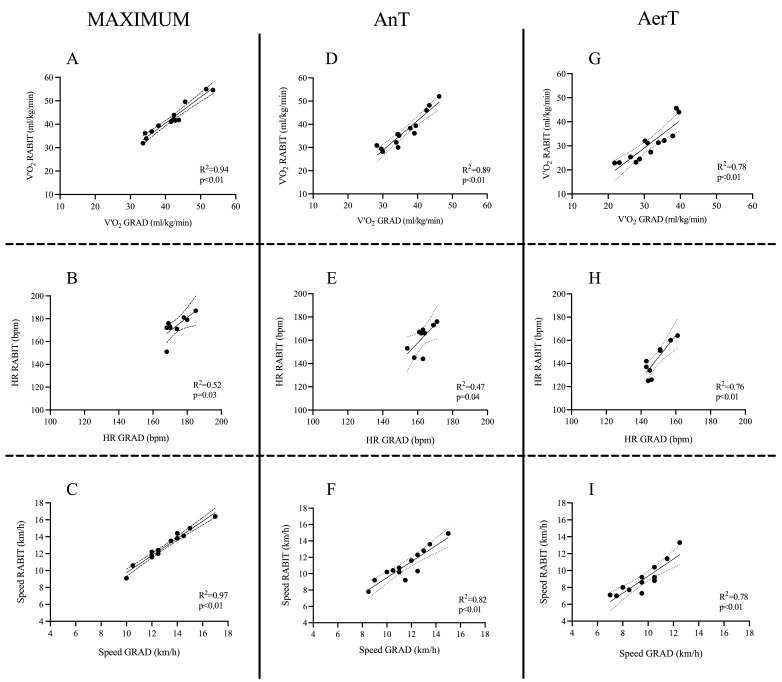
Correlation and confidence intervals (95%) between the results of RABIT^®^ vs. GRAD in the cardiorespiratory parameters at three intensities (maximum: (**A**–**C**); AnT: (**D**–**F**); AerT: (**G**–**I**)). $\dot{V}$O_2_: oxygen uptake; HR: heart rate; AerT: aerobic threshold; AnT: anaerobic threshold.

**Figure 3:**
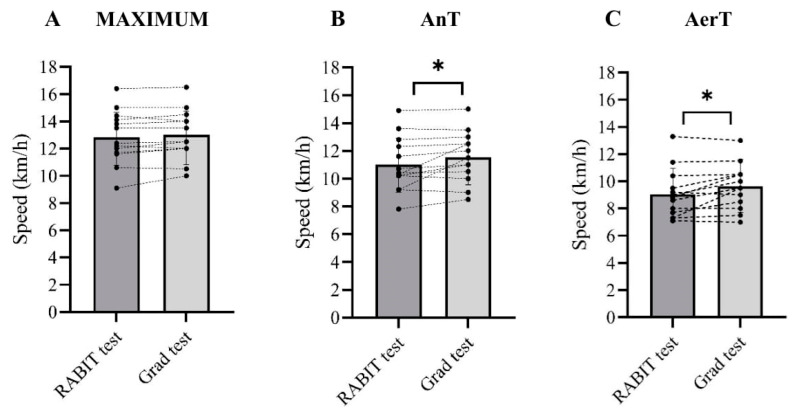
Individual differences between the speed reached during the RABIT^®^ and GRAD at three intensities: RPE 18 and maximum (panel (**A**)), RPE 16 and AnT (panel (**B**)), and RPE 13 and AerT (panel (**C**)). * *p* < 0.05. RPE: rating of perceived exertion; AerT: aerobic threshold; AnT: anaerobic threshold.

At RPE 18, the values of $\dot{V}$O_2_max, $\dot{V}$_E_, HRmax, and maximal running speed were similar between RABIT^®^ and the GRAD (Table 1; Figure 2A–C and Figure 3A). RER max at RPE 18 of the RABIT^®^ test was lower by −3.78% (*p* < 0.001) compared to GRAD. Linear regression between GRAD and RABIT^®^ for $\dot{V}$O_2_max, HRmax, and maximal running speed was R^2^ = 0.94 (*p* < 0.001), R^2^ = 0.52 (*p* < 0.030), and R^2^ = 0.97 (*p* < 0.001), respectively. The accurate prediction was 85% for $\dot{V}$O_2_max, 82% for RERmax, 54% for minute ventilation, 93% for HRmax, and 92% for running speed. The ES and 95% confidence intervals (95% CI) were established for $\dot{V}$O_2_max (ES: 0.09 *small*; 95% CI: −0.50 to 1.75), RERmax (ES: 1.56 *large*; 95% CI: −0.06 to −0.02), $\dot{V}$_E_ (ES: 0.20 *medium*; 95% CI: −8.50 to 2.43), HRmax (ES: 0.09 *small*; 95% CI: −5.71 to 5.30), and maximal running speed (ES: 0.10 *small*; 95% CI: −0.42 to 0.01).

### 3.2. Anaerobic Threshold

**Figure 4:**
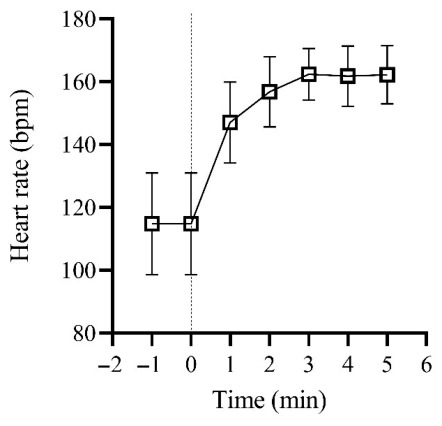
Mean (±SD) values of heart rate (HR) during the RABIT^®^ test at RPE 16. Vertical lines indicate that exercise started at time 0. See results for further details.

At RPE 16, $\dot{V}$O_2_, RER, $\dot{V}$_E,_ and HR measured during the RABIT^®^ test were not significantly different from the corresponding parameters obtained at AnT during the GRAD (Table 1 and Figure 2D,E). The running speed was significantly lower in the RABIT^®^ test in comparison to GRAD (−5.03%, *p* = 0.041) (Figure 2F and Figure 3B). Linear regression between GRAD and RABIT^®^ tests for $\dot{V}$O_2_, HR, and running speed were R^2^ = 0.89 (*p* < 0.001), R^2^ = 0.47 (*p* < 0.0040), and R^2^ = 0.82 (*p* < 0.001), respectively. The accurate prediction was 62% for $\dot{V}$O_2_, 67% for RER, 46% for $\dot{V}$_E_, 78% for HR, and 77% for running speed. The ES and 95% CI were established for $\dot{V}$O_2_ (ES: 0.07 *small*; 95% CI: −1.21 to 2.40), RER (ES: 0.33 *medium*; 95% CI: −0.05 to 0.01), $\dot{V}$_E_ (ES: 0.48 *medium*; 95% CI: −15.2 to 2.40), HR (ES: 0.11 *small*; 95% CI: −7.65 to 6.31) and running speed (ES: 0.26 *medium*; 95% CI: 1.02 to −0.02). During RPE 16, the slope of the HR versus time regression lines was not significantly different between minutes four and five (*p* = 0.44) (Figure 4).

### 3.3. Aerobic Threshold

At RPE 13, the values of $\dot{V}$O_2_, RER, $\dot{V}$_E,_ and HR were similar between the RABIT^®^ and AerT during the GRAD (Table 1 and Figure 2G,H). The running speed during the RABIT^®^ test was significantly lower than the speed at AerT measured during GRAD (−7.00%, *p* < 0.001, Figure 2I and Figure 3C). Linear regression between GRAD and RABIT^®^ tests for $\dot{V}$O_2_, HR, and running speed were R^2^ = 0.78 (*p* < 0.001), R^2^ = 0.76 (*p* < 0.001), and R^2^ = 0.78 (*p* < 0.001), respectively. The accurate prediction was 46% for $\dot{V}$O_2_, 91% for RER, 46% for $\dot{V}$_E_, 67% for HR, and 62% for running speed. The ES and 95% CI were established for $\dot{V}$O_2_ (ES: 0.12 *small*; 95% CI: –2.87 to 1.43), RER (ES: 0.01 *small*; 95% CI: −0.03 to 0.01), $\dot{V}$_E_ (ES: 0.32 *medium*; 95% CI: −8.53 to 3.54), HR (ES: 0.01 *small*; 95% CI: −12.54 to 1.43), and running speed (ES: 0.28 *medium*; 95%CI: −1.04 to −0.10).

## 4. Discussion

In the present study, we observed that the modified RABIT^®^ test, when applied to adults with obesity, demonstrated: (1) significant concordance with the GRAD for maximal intensity parameters, with no significant differences between methods; (2) comparable results for AnT-related parameters; and (3) comparable results for AerT-related parameters, although the RABIT^®^ test yielded significantly lower values for running speed only.

***Maximum***

At RPE 18, $\dot{V}$O_2_max, $\dot{V}$E, HRmax, and maximal running speed obtained during the RABIT^®^ test were comparable to those measured during the GRAD, as confirmed by the absence of significant differences and the strong correlations observed between the parameters collected in the two tests. A significant difference was detected only for RERmax, which was lower in RABIT^®^ by −3.78 ± 3.19% (*p* < 0.001) compared to GRAD. It is possible that individuals with obesity, during high-intensity efforts such as the 3 min stage at RPE 18, exhibit a reduced capacity for CO_2_ elimination [36], potentially due to altered ventilatory efficiency and mechanical constraints on the respiratory system. These factors may have contributed to the slightly lower RER values observed in the RABIT^®^ conditions. From a physiological perspective, RPE reflects the integration of central and peripheral signals arising from the cardiovascular, respiratory, and muscular systems [37,38]. As exercise intensity approaches $\dot{V}$O_2_max, increased motor unit recruitment, metabolite accumulation, and ventilatory drive amplify afferent feedback from working muscles and cardiorespiratory structures, thereby increasing the conscious perception of effort [39,40]. From a practical standpoint, HR and speed derived from the RABIT^®^ test at maximal intensity appear suitable for exercise prescription. At RPE 18, running speed and HR were only ~2% and ~0.5% lower, respectively, than the values recorded during the GRAD, indicating a high level of agreement at peak effort. Moreover, the 95% limits of agreement for HRmax (± 8%) reflect the expected inter-individual variability in heart rate response to strenuous exercise in adults with obesity and remain within an acceptable range for high-intensity interval training prescription in a field setting [41,42]. Overall, these preliminary results support that, at high perceived exertion levels, RPE can be meaningfully anchored to objective physiological parameters such as $\dot{V}$O_2_max, HRmax, and maximal running speed. This strengthens its utility as a practical and scalable tool for both performance assessment and training prescription in adults with obesity.

***Anaerobic threshold***

At RPE 16, $\dot{V}$O_2_, RER, $\dot{V}$E, and HR measured during the RABIT^®^ test did not significantly differ from the values obtained at AnT during the GRAD, supporting the potential validity of a 5 min stage at RPE 16 for identifying the heavy domain (e.g., z2) in adults with obesity. Overall, these results indicate that the internal physiological load was comparable between the two protocols. In contrast, running speed at AnT was ~5% lower (~0.5 km·h^−1^) during RABIT^®^ compared with GRAD, despite a predictive accuracy of ~80% and a strong correlation between the two protocols. Notably, this ~5% difference is slightly greater than the ~3% previously reported in endurance athletes [18]. The lower running speed observed during RABIT^®^ suggests that exercise intensities derived from an incremental test, such as GRAD, may not be fully sustainable under constant-load conditions near the heavy domain. During constant-load exercise, progressive metabolite accumulation, increased ventilatory drive, and nociceptive input likely elevate perceived exertion and influence pacing strategies, leading to a voluntary downregulation of running speed to maintain a tolerable effort [43,44]. This adjustment is consistent with findings from RPE-controlled exercise, where power output or speed has been shown to decrease within the first 5–7 min, with reductions of ~8% during cycling compared with the intensity reached at the same relative effort during an incremental test [45,46,47]. At the same time, although speeds obtained during a GRAD are not always directly comparable with those sustained during prolonged constant-load exercise, the difference observed between the two protocols in the present study falls within the absolute error typically reported for AnT speed when assessed in two closely repeated tests (bias < 0.5 km·h^−1^) [48]. Therefore, the magnitude of the discrepancy should also be interpreted considering the inherent measurement variability. Moreover, HR responses did not significantly differ between RABIT^®^ and GRAD, suggesting that HR at RPE 16 can be effectively used to monitor and prescribe steady-state training sessions lasting 30–60 min or long-interval training. When comparing HR at the end of the fourth and fifth minute of RABIT^®^ at RPE 16, no HR slow component was detected (*p* = 0.44). In contrast, Zuccarelli et al. [32] reported the presence of an HR slow component above AerT in patients with severe obesity (BMI ≥ 40 kg·m^−2^). It is plausible that the lower degree of obesity and the relatively better cardiorespiratory fitness of our sample facilitated the attainment of a stable physiological response close to AnT. Taken together, these findings support HR at RPE 16 as a valid and practical indicator of the AnT domain for exercise prescription in adults with obesity.

***Aerobic threshold***

At RPE 13, the physiological responses measured during the RABIT^®^ test were similar to those observed at AerT during the GRAD, confirming the usefulness of the 10 min stage of the RABIT^®^ test for identifying the moderate-intensity domain (e.g., z1) in individuals with obesity. However, running speed was ~7% lower in RABIT^®^ compared with AerT derived from GRAD, despite a reasonably accurate prediction (~60%) and a high correlation. Previous studies showed similar differences in running speed measured during the 10 min stage of the RABIT^®^ and running speed at AerT during GRAD in well-trained runners [18]. This discrepancy should be interpreted as the fact that, during GRAD, participants ran only one minute through the intensity corresponding to AerT without reaching a metabolic steady state in which homeostatic regulation, ventilatory patterns, and pacing strategies can stabilize [49]. This suggests that, during longer submaximal exercise performed under self-paced and RPE-clamped conditions, individuals adjust the external load in response to internal signals, such as respiratory effort and thermal discomfort, as previously reported in the literature [50]. Regarding HR, we observed similar values between the two tests, with higher predictive accuracy (~70%) and a stronger correlation compared with running speed. These findings suggest that determining HR at AerT using the RABIT^®^ test at this intensity may be useful for exercise prescription. Indeed, in individuals with obesity, especially during the initial phases of training, it may be preferable to prescribe exercise intensity primarily based on HR rather than external measures such as running speed, which can be influenced by environmental and biomechanical factors [51]. Further support of this comes from a recent study by Novak et al. [52], showing that adherence to prescribed HR zones was a significant predictor of improvements in $\dot{V}$O_2_max in sedentary adults. In this context, the RABIT^®^ test may represent a practical and valid tool for initial assessment and for establishing individualized training zones based on estimated HR and speed at ventilatory thresholds, rather than relying on fixed percentages of HRmax, which may limit improvements and fail to account for individual variability in aerobic exercise responses [53]. However, because HR data were available for only 9 of the 13 participants, these findings, although promising, should be interpreted with caution. A larger sample size and higher predictive accuracy would provide stronger confirmation of our results.

### Limitations

The present study has some limitations. The sample size was relatively small and included only male participants with obesity, which limits the generalizability of the findings to normal-weight or overweight male adults, as well as females. Second, although our data showed acceptable preliminary evidence supporting validity, the inclusion of additional familiarization trials could further improve the reliability and reproducibility of performance indices obtained during the self-regulated RPE test. Third, while the predictive accuracy of speed at RPE 13 and RPE 16 was promising, the observed underestimation of running speed compared with the GRAD suggests that caution should be exercised when using RABIT^®^-derived velocities for training prescription.

## 5. Conclusions

The results of the present study suggest that the RABIT^®^ test could represent a simple, low-cost field method for detecting parameters (i.e., speed and HR) associated with exercise training zones outside laboratory settings. Overall, the findings showed a moderate level of agreement with the GRAD, particularly for $\dot{V}$O_2_ and HR. However, relevant discrepancies were observed for RER and running speed, and some variability in predictive accuracy should be acknowledged. Therefore, the RABIT^®^ test should be interpreted as a practical supportive tool rather than a replacement for laboratory-based assessment, and for training optimization, we recommend combining HR and running speed to obtain more accurate and individualized information. Further research is warranted to confirm its preliminary evidence supporting validity and to explore its responsiveness to training-induced adaptations over time.

## Figures and Tables

**Table 1:** Parameters obtained during the RABIT^®^ test and GRAD.

	RABIT Test	GRAD	Differences (%)	95% Limits of Agreement		Accurate Prediction	*p*
**Maximum—RPE 18**							
Oxygen uptake (mL/kg/min)	42.1 ± 7.2	41.5 ± 6.3	1.2 ± 4.3	−7.2	9.6	85	0.258
Respiratory exchange ratio	1.05 ± 0.02	1.09 ± 0.03	−3.6 ± 3.1	−9.6	2.3	82	0.001
Minute ventilation (L/min)	126.7 ± 16.3	123.6 ± 13.5	2.2 ± 7.6	−12.5	17.1	54	0.250
Heart rate (bpm)	173 ± 10	172 ± 7	−0.2 ± 4.3	−8.5	8.3	93	0.964
Speed (km/h)	12.8 ± 2.0	13.0 ± 1.8	−1.7 ± 3.1	−7.9	4.3	92	0.060
**Anaerobic threshold—RPE 16**							
Oxygen uptake (mL/kg/min)	37.0 ± 7.6	36.5 ± 5.6	−1 ± 7.7	−5.3	6.5	62	0.489
Respiratory exchange ratio	0.98 ± 0.03	0.99 ± 0.03	−2.1 ± 5.2	−0.1	0.08	67	0.194
Minute ventilation (L/min)	103.8 ± 15.3	97.4 ± 11.0	5.9 ± 15.0	−23.4	35.3	46	0.140
Heart rate (bpm)	162 ± 12	161 ± 6	−0.6 ± 5.8	−18.5	17.1	78	0.831
Speed (km/h)	11.0 ± 2.0	11.5 ± 1.8	−4.9 ± 7.7	−2.1	1.1	77	0.041
**Aerobic threshold—RPE 13**							
Oxygen uptake (mL/kg/min)	30.5 ± 7.5	31.3 ± 5.8	−3.2 ± 10.6	−7.7	6.3	46	0.478
Respiratory exchange ratio	0.91 ± 0.03	0.91 ± 0.04	−0.1 ± 3.7	−0.07	0.05	91	0.337
Minute ventilation (L/min)	75.2 ± 8.0	72.8 ± 6.9	4.3 ± 13.5	−15.6	22.1	46	0.386
Heart rate (bpm)	143 ± 14	143 ± 7	−4.1 ± 6.6	−23.4	12.3	67	0.104
Speed (km/h)	9.00 ± 1.82	9.60 ± 1.70	−7.6 ± 9.2	−2.4	1.0	62	0.020

All values are expressed as mean ± standard deviation. ^1^ Accurate prediction: percentage of all subjects whose RABIT^®^ parameters were within 95% to 105% of GRAD parameters.

## Data Availability

The data supporting this publication are available from the authors upon reasonable request.

## Abbreviations

The following abbreviations are used in this manuscript:

RABIT^®^	Running Advisor Billat Training test
GRAD	Gaded exercise test
RPE	Rating of perceived exertion
$\dot{V}$O_2_max	Maximal oxygen consumption
$\dot{V}$O_2_	Oxygen uptake
$\dot{V}$CO_2_	Carbon dioxide production
$\dot{V}$_E_	Minute ventilation
HR	Heart rate
HRmax	Maximal heart rate
AerT	Aerobic threshold
AnT	Anaerobic threshold
IPAQ-SF	International Physical Activity Questionnaire Short Form
CS	Critical speed
BM	Body mass
BMI	Body mass index
FM	Fat mass
RER	Respiratory exchange ratio

## References

[1] World Health Organization Obesity and Overweight. https://www.who.int/news-room/fact-sheets/detail/obesity-and-overweight.

[2] Bull F.C., Al-Ansari S.S., Biddle S., Borodulin K., Buman M.P., Cardon G., Carty C., Chaput J.-P., Chastin S., Chou R. (2020). World Health Organization 2020 guidelines on physical activity and sedentary behaviour. Br. J. Sports Med..

[3] Jepsen R., Aadland E., Robertson L., Kolotkin R.L., Andersen J.R., Natvig G.K. (2015). Physical Activity and Quality of Life in Severely Obese Adults during a Two-Year Lifestyle Intervention Programme. J. Obes..

[4] Wang T., Yang L., Xu Q., Dou J., Clemente F.M. (2024). Effects of recreational team sports on the metabolic health, body composition and physical fitness parameters of overweight and obese populations: A systematic review. Biol. Sport.

[5] Kutac P., Bunc V., Buzga M., Krajcigr M., Sigmund M. (2023). The effect of regular running on body weight and fat tissue of individuals aged 18 to 65. J. Physiol. Anthropol..

[6] Lee D., Brellenthin A.G., Thompson P.D., Sui X., Lee I.-M., Lavie C.J. (2017). Running as a Key Lifestyle Medicine for Longevity. Prog. Cardiovasc. Dis..

[7] Vincent H.K., Kilgore J.E., Chen C., Bruner M., Horodyski M., Vincent K.R. (2020). Impact of Body Mass Index on Biomechanics of Recreational Runners. PMR.

[8] Jamnick N.A., Pettitt R.W., Granata C., Pyne D.B., Bishop D.J. (2020). An Examination and Critique of Current Methods to Determine Exercise Intensity. Sports Med..

[9] Thompson M.A. (2017). Physiological and Biomechanical Mechanisms of Distance Specific Human Running Performance. Integr. Comp. Biol..

[10] Beltz N.M., Gibson A.L., Janot J.M., Kravitz L., Mermier C.M., Dalleck L.C. (2016). Graded Exercise Testing Protocols for the Determination of VO2max: Historical Perspectives, Progress, and Future Considerations. J. Sports Med..

[11] Seiler S. (2010). What is Best Practice for Training Intensity and Duration Distribution in Endurance Athletes?. Int. J. Sports Physiol. Perform..

[12] Stöggl T.L., Sperlich B. (2015). The training intensity distribution among well-trained and elite endurance athletes. Front. Physiol..

[13] Billat V., Dalmay F., Antonini M.T., Chassain A.P. (1994). A method for determining the maximal steady state of blood lactate concentration from two levels of submaximal exercise. Eur. J. Appl. Physiol..

[14] Cooper K.H. (1968). A Means of Assessing Maximal Oxygen Intake: Correlation Between Field and Treadmill Testing. JAMA.

[15] Galbraith A., Hopker J., Lelliott S., Diddams L., Passfield L. (2014). A Single-Visit Field Test of Critical Speed. Int. J. Sports Physiol. Perform..

[16] Forte L.D.M., Manchado-Gobatto F.B., Rodrigues R.C.M., Gallani M.C., Gobatto C.A. (2018). Non-exhaustive double effort test is reliable and estimates the first ventilatory threshold intensity in running exercise. J. Sport Health Sci..

[17] Molinari C.A., Palacin F., Poinsard L., Billat V.L. (2020). Determination of Submaximal and Maximal Training Zones From a 3-Stage, Variable-Duration, Perceptually Regulated Track Test. Int. J. Sports Physiol. Perform..

[18] Giovanelli N., Scaini S., Billat V., Lazzer S. (2020). A new field test to estimate the aerobic and anaerobic thresholds and maximum parameters. Eur. J. Sport Sci..

[19] Borg G. (1970). Perceived exertion as an indicator of somatic stress. Scand. J. Rehabil. Med..

[20] Bok D., Rakovac M., Foster C. (2022). An Examination and Critique of Subjective Methods to Determine Exercise Intensity: The Talk Test, Feeling Scale, and Rating of Perceived Exertion. Sports Med..

[21] Lopes T.R., Pereira H.M., Silva B.M. (2022). Perceived Exertion: Revisiting the History and Updating the Neurophysiology and the Practical Applications. Int. J. Environ. Res. Public Health.

[22] Ekkekakis P., Parfitt G., Petruzzello S.J. (2011). The pleasure and displeasure people feel when they exercise at different intensities: Decennial update and progress towards a tripartite rationale for exercise intensity prescription. Sports Med..

[23] Lafortuna C.L., Agosti F., Galli R., Busti C., Lazzer S., Sartorio A. (2008). The energetic and cardiovascular response to treadmill walking and cycle ergometer exercise in obese women. Eur. J. Appl. Physiol..

[24] Coquart J.-B., Tourny-Chollet C., Lemaître F., Lemaire C., Grosbois J.-M., Garcin M. (2012). Relevance of the measure of perceived exertion for the rehabilitation of obese patients. Ann. Phys. Rehabil. Med..

[25] Hydren J.R., Cohen B.S. (2015). Current Scientific Evidence for a Polarized Cardiovascular Endurance Training Model. J. Strength Cond. Res..

[26] Wood R.E., Hills A.P., Hunter G.R., King N.A., Byrne N.M. (2010). $\dot{V}$O_2_max in Overweight and Obese Adults: Do They Meet the Threshold Criteria?. Med. Sci. Sports Exerc..

[27] Craig C.L., Marshall A.L., Sjöström M., Bauman A.E., Booth M.L., Ainsworth B.E., Pratt M., Ekelund U., Yngve A., Sallis J.F. (2003). International Physical Activity Questionnaire: 12-Country Reliability and Validity. Med. Sci. Sports Exerc..

[28] Hoogkamer W., Kipp S., Spiering B.A., Kram R. (2016). Altered Running Economy Directly Translates to Altered Distance-Running Performance. Med. Sci. Sports Exerc..

[29] D’Alleva M., Vaccari F., Graniero F., Giovanelli N., Floreani M., Fiori F., Marinoni M., Parpinel M., Lazzer S. (2023). Effects of 12-week combined training versus high intensity interval training on cardiorespiratory fitness, body composition and fat metabolism in obese male adults. J. Exerc. Sci. Fit..

[30] Beaver W.L., Wasserman K., Whipp B.J. (1986). A new method for detecting anaerobic threshold by gas exchange. J. Appl. Physiol..

[31] Howley E.T., Bassett D.R., Welch H.G. (1995). Criteria for maximal oxygen uptake: Review and commentary. Med. Sci. Sports Exerc..

[32] Zuccarelli L., Porcelli S., Rasica L., Marzorati M., Grassi B. (2018). Comparison between Slow Components of HR and V˙O_2_ Kinetics: Functional Significance. Med. Sci. Sports Exerc..

[33] Bland J.M., Altman D.G. (1986). Statistical methods for assessing agreement between two methods of clinical measurement. Lancet.

[34] Atkinson G., Nevill A.M. (1998). Statistical Methods For Assessing Measurement Error (Reliability) in Variables Relevant to Sports Medicine. Sports Med..

[35] Phang P.T., Rich T., Ronco J. (1990). A Validation and Comparison Study of Two Metabolic Monitors. J. Parenter. Enter. Nutr..

[36] Balmain B.N., Halverson Q.M., Tomlinson A.R., Edwards T., Ganio M.S., Babb T.G. (2021). Obesity Blunts the Ventilatory Response to Exercise in Men and Women. Ann. Am. Thorac. Soc..

[37] Borg G.A. (1982). Psychophysical bases of perceived exertion. Med. Sci. Sports Exerc..

[38] Noble B.J., Robertson R.J. (1996). Perceived Exertion.

[39] Marcora S. (2009). Perception of effort during exercise is independent of afferent feedback from skeletal muscles, heart, and lungs. J. Appl. Physiol..

[40] Amann M., Secher N.H. (2010). Point: Afferent feedback from fatigued locomotor muscles is an important determinant of endurance exercise performance. J. Appl. Physiol..

[41] Lanzi S., Codecasa F., Cornacchia M., Maestrini S., Capodaglio P., Brunani A., Fanari P., Salvadori A., Malatesta D. (2015). Long Maximal Incremental Tests Accurately Assess Aerobic Fitness in Class II and III Obese Men. PLoS ONE.

[42] Boulay P., Ghachem A., Poirier P., Sigal R.J., Kenny G.P. (2025). Assessment of Maximum Heart Rate Prediction Equations in Adults at Low and High Risk of Cardiovascular Disease. Med. Sci. Sports Exerc..

[43] Mauger L. (2014). Factors affecting the regulation of pacing: Current perspectives. Open Access J. Sports Med..

[44] Black M.I., Jones A.M., Blackwell J.R., Bailey S.J., Wylie L.J., McDonagh S.T.J., Thompson C., Kelly J., Sumners P., Mileva K.N. (2017). Muscle metabolic and neuromuscular determinants of fatigue during cycling in different exercise intensity domains. J. Appl. Physiol..

[45] Cochrane-Snyman K.C., Housh T.J., Smith C.M., Hill E.C., Jenkins N.D.M. (2019). Treadmill running using an RPE-clamp model: Mediators of perception and implications for exercise prescription. Eur. J. Appl. Physiol..

[46] O’Malley C.A., Fullerton C.L., Mauger A.R. (2023). Test–retest reliability of a 30-min fixed perceived effort cycling exercise. Eur. J. Appl. Physiol..

[47] O’Grady C., Passfield L., Hopker J.G. (2021). Variability in Submaximal Self-Paced Exercise Bouts of Different Intensity and Duration. Int. J. Sports Physiol. Perform..

[48] Cerezuela-Espejo V., Courel-Ibáñez J., Morán-Navarro R., Martínez-Cava A., Pallarés J.G. (2018). The Relationship Between Lactate and Ventilatory Thresholds in Runners: Validity and Reliability of Exercise Test Performance Parameters. Front. Physiol..

[49] Poole D.C., Jones A.M. (2012). Oxygen Uptake Kinetics. Compr. Physiol..

[50] Nguyen A.P., Kisita V., Van Cant J., Monnet T., Bosquet L. (2025). Reproducibility of Rate of Perceived Exertion–Based Self-Selected Running Speeds on Indoor Track and Treadmill Conditions in Recreational Runners. J. Strength Cond. Res..

[51] Achten J., Jeukendrup A.E. (2003). Heart Rate Monitoring: Applications and Limitations. Sports Med..

[52] Novak T.S., Quan C., McGregor K., Mammino K., Bello M., Nocera J.R. (2026). Adherence to heart rate-based intensity parameters predicts cardiovascular response to 12-weeks of aerobic cycling training in sedentary older adults. Prev. Med. Rep..

[53] Anselmi F., Cavigli L., Pagliaro A., Valente S., Valentini F., Cameli M., Focardi M., Mochi N., Dendale P., Hansen D. (2021). The importance of ventilatory thresholds to define aerobic exercise intensity in cardiac patients and healthy subjects. Scand. J. Med. Sci. Sports.

